# Dissecting the Molecular Function of *Triticum aestivum STI* Family Members Under Heat Stress

**DOI:** 10.3389/fgene.2020.00873

**Published:** 2020-08-19

**Authors:** Shaloo Meena, Sohini Deb, Harsha Samtani, Paramjit Khurana

**Affiliations:** Department of Plant Molecular Biology, University of Delhi South Campus, New Delhi, India

**Keywords:** co-chaperone, STI, heat stress, thermotolerance, endoplasmic reticulum (ER)

## Abstract

STI/HOP functions as a co-chaperone of HSP90 and HSP70 whose molecular function has largely been being restricted as an adaptor protein. However, its role in thermotolerance is not well explored. In this article, we have identified six members of the *TaSTI* family, which were named according to their distribution on group 2 and group 6 chromosomes. Interestingly, *TaSTI-2* members were found to express higher as compared to *TaSTI-6* members under heat stress conditions, with *TaSTI-2A* being one of the most heat-responsive member. Consistent with this, the heterologous expression of *TaSTI-2A* in *Arabidopsis* resulted in enhanced basal as well as acquired thermotolerance as revealed by the higher yield of the plants under stress conditions. Similarly in the case of rice, *TaSTI-2A* transgenics exhibited enhanced thermal tolerance. Moreover, we demonstrate that TaSTI-2A interacts with TaHSP90 not only in the nucleus but also in the ER and Golgi bodies, which has not been shown till now. Additionally, TaHSP70 was also found to interact with TaSTI-6D specifically in the cytosol. Thus, these data together suggested that the *TaSTI* family members might play different roles under heat stress conditions in order to fine-tune the heat stress response in plants.

## Introduction

Plants unlike animals cannot escape from unfavorable environmental conditions. Changes in ambient temperature become the most common form of stress encountered by plants. When the temperature reaches higher than that required for optimum growth, it leads to irreversible damage and is generally known as heat stress (HS) (Wahid et al., 2007). At the cellular level, HS causes the accumulation of misfolded proteins, which not only leads to loss in activity but also causes protein aggregation, thereby disturbing cell homeostasis (Nakajima and Suzuki, 2013). Thus, cells are equipped with a Quality Control (QC) system consisting of chaperones and degradative complexes. The QC system not only helps in the folding of the newly synthesized proteins but also recognizes the misfolded proteins and facilitates their refolding. However, if any protein is unable to fold correctly, then it is targeted for degradation (Buchberger et al., 2010; Houck et al., 2012). In plants, the expression of these chaperones is enhanced by a conserved mechanism known as heat stress response (HSR) (Hahn et al., 2011). Among the different chaperones present in the cell, HSP70 and HSP90 play a major role in QC in the cytoplasm under HS conditions. HSP70 accumulates abundantly in the cells and binds to the hydrophobic patches of the misfolded proteins in an ATP-dependent manner, thereby preventing their aggregation (Mayer and Bukau, 2005; Hahn et al., 2011), whereas HSP90 has been found to regulate the activity of different transcription factors, kinases and steroid hormone receptors (Hahn et al., 2011). The activity of these chaperones is regulated by the binding of different co-chaperones that modulate their structural conformation and ATPase activity and, hence, the substrate selection (Li et al., 2012; Prodromou, 2012).

The HSP90/70 organizing protein (HOP) is the co-chaperone that functions as an adaptor in HSP90 and HSP70 machinery (Chen and Smith, 1998; Scheufler et al., 2000; Chen et al., 2010). HOP, also known as STI (stress-induced protein), was first identified in yeast during a genetic screen for proteins involved in HSR (Nicolet and Craig, 1989). Unlike HSP90 and HSP70, STI does not possess any chaperone activity (Bose et al., 1996; Freeman et al., 1996), but it consists of several tetratricopeptide repeat (TPR) domains which are involved in protein–protein interactions. The N-terminus of the TPR1 domain binds to the C-terminus of HSP70, and the central TPR2A domain binds to the C-terminus of HSP90 (Scheufler et al., 2000; Hernandez et al., 2002).

Various functional roles of STI has been reported in animals (Baindur-Hudson et al., 2015). STI has been isolated and characterized in humans as transformation-sensitive human protein (IEF-SSP3521), and its potential role has been suggested in cell proliferation or gene regulation (Honore et al., 1992). STI also interacts with cellular prions and is involved in neuritogenesis and leads to neuroprotection (Zanata et al., 2002; Lopes et al., 2005). In the case of *C. elegans*, the expression of *STI1* was found to be induced by HS. Also, the mutants of *STI1* in *C. elegans* showed a shorter life span and decreased fertility rate, thereby indicating its role in stress responses and in aging processes (Song et al., 2009). A recent report by Karam et al. has highlighted the unique role of STI in transposon silencing and Piwi-interacting RNA biogenesis (Karam et al., 2017).

In the case of plants, *STI* homologs have been described in *Arabidopsis thaliana, Glycine max*, and *Oryza sativa* (Zhang et al., 2003; Fellerer et al., 2011; Fernandez-Bautista et al., 2018). In *Arabidopsis*, the role of HOP has been described in alleviating ER stress response (Fernandez-Bautista et al., 2017). Also, STI was found as one of the partner proteins in HSP90-chloroplast preprotein complex, indicating its role in chaperoning preproteins in plant cytosol (Fellerer et al., 2011). Interestingly, many studies have uncovered its role in plant-pathogen defense responses. STI, along with HSP90, has been shown to interact with rice chitin receptor (OsCERK1) and is involved in innate immunity response against rice blast fungus (Chen et al., 2010). Similarly, STI has been found to act as a major cellular determinant for the mitochondrial *Carnation Italian Ringspot Tombusvirus* (CIRV) and the *Potato Virus Y* proliferation in *Nicotiana benthamiana* and tobacco, respectively (Xu et al., 2014; Lamm et al., 2017). Apart from these, the function of *STI* in various abiotic stresses still remains unclear. A recent study has shown the role of *Arabidopsis STI* family in long term-acquired thermotolerance (Fernandez-Bautista et al., 2018). In the case of *Triticum aestivum, STI* was found to be induced by HS (Chauhan et al., 2011). Thus, these evidences suggest that STI might have broader functions rather than being merely an adaptor protein.

In this study, we have identified six members of *STI* from wheat and analyzed their possible role under HS. Expression profiling of these members revealed their differential regulation, and their localization studies showed their presence in the nucleus, cytoplasm, and ER-Golgi complex. Interestingly, one of the most heat-responsive members, TaSTI-2A, was found to interact with TaHSP90 in the nucleus as well as in the ER and Golgi bodies. TaHSP70 was also found to interact with a different member of the STI gene family, i.e., TaSTI-6D in the cytosol, which has not yet been reported in the case of plants. The overexpression of *TaSTI-2A* promoted thermotolerance in both *A. thaliana* and *O. sativa*. Thus, our results suggest that *TaSTI* could serve as a potential gene for heat tolerance enhancement in crops.

## Materials and Methods

### Identification and Structural Analysis of *STI* Gene Family Members From Wheat

STI protein sequences of *A. thaliana* were taken from the TAIR database (AT1G12270, AT1G62740, AT4G12400). These protein sequences were further used for Blast search against available *T. aestivum* genome (*T. aestivum*-Ensembl Genomes 41), and a total of six members were identified. One of the TaSTI members was previously identified in the lab from the HS cDNA library (GD189073), which was submitted to the NCBI database (Chauhan et al., 2011). This STI member was also found in the Ensembl database, and we have named it as TaSTI-2A. These protein sequences were checked for conserved STI and TPR domains using the NCBI CDD search and SMART domain tool analysis. Multiple sequence alignment was carried out by the CLUSTALW program, and the phylogenetic tree was constructed using the MEGA7 program by Neighbor-Joining method.

Moreover, the Phyre2 web portal was used for the prediction of the three-dimensional structure of *Ta*STI and *At*HOP proteins.

### Expression Analysis of *TaSTI* Members in Different Genotypes of Wheat and in *A. thaliana*

Bread wheat (*T. aestivum*) cultivar PBW343 and C306 and *Arabidopsis* ecotype Col-0 were used in this study. Wheat seeds were surface-sterilized with 4% sodium hypochlorite for 20 min followed by five to six washes with autoclaved sterile water. After sterilization, seeds were grown on a cotton tray in a growth chamber (Conviron, Canada) maintained at 22 ± 1°C with a 16-h photoperiod. *Arabidopsis* plants were grown on half strength Murashige and Skoog (MS) media. For the stress treatment of wheat, 10-day-old seedlings were subjected to different abiotic stresses such as heat (42°C for 2 h), cold (4°C for 24 h), salt (200 mM for 24 h), and drought (200 mM mannitol for 24 h) (Peng et al., 2009; Chauhan et al., 2011; Amoah et al., 2019; Hamdi et al., 2020). Ten-day-old seedlings of genotype PBW343 and C306 were subjected to different temperatures ranging from 25°C to 45°C. After the stress treatment, seedlings of control and treated plants were frozen in liquid nitrogen and stored at −80°C until RNA isolation. *Arabidopsis* plants were germinated on half MS media and then transferred to soilrite for further analysis.

Total RNA was isolated using the RNeasy plant mini kit (Qiagen, Germany) according to the manufacturer’s instructions, including on-column DNaseI treatment to remove genomic DNA contamination. Two micrograms of the total RNA was used as a template to synthesize cDNA employing the High Capacity cDNA Archive kit (Applied Biosystems, United States) and mixed with 200 nM of each primer and SYBR Green PCR Master Mix (Applied Biosystems) for real-time PCR analysis, using the ABI Prism 7000 Sequence Detection System and Software (PE Applied Biosystems) according to the manufacturer’s protocol. Relative fold change was calculated, and *Actin* was used as a housekeeping gene. Graphs were plotted using three biological and three technical replicates. The wheat expression database (Borrill et al., 2016) hosted at http://wheatexpression.com was used to analyze the expression profile of the *STI* members. This database was developed as an expression visualization and integration platform. Further, this database also hosts the normalized data for many development and stress treated wheat samples. Therefore, transcripts per kilobase million (TPM) values for all the *STI* members were downloaded from this database. TPM values were log-transformed (Log2X) in order to generate a heatmap using the gplots package and Rcolorbrewer package.

### *Cis* Element Analysis of TaSTI Promoter Sequences

For the analysis of URRs (upstream regulatory regions), the 2-kb upstream region of all the *TaSTI* members was extracted from the Ensembl database (*T. aestivum*-Ensembl Genomes 41). The *cis*-elements in promoters were subsequently searched using the PLACE and PlantCare database (Higo et al., 1999; Lescot et al., 2002).

### Yeast-2-Hybrid Assay

For the measurement of the interaction, the putative interactants, *TaSTI1-2A* and *TaHSP90* were cloned into pENTR/D-TOPO vectors and further into pDEST-GADT7 and pDEST-GBKT7 vectors (Clontech, United States). The recombinant plasmids were transformed into the yeast strain AH109 harboring the ADE3 and HIS3 reporter genes. The reporter gene activity was confirmed by a viability test on a medium lacking histidine, leucine, and tryptophan (-HLW) along with 0.5 mM 3-AT (3-Amino-1,2,4-triazole).

### Complementation Assay

To check for complementation, the full-length *TaSTI*-2A gene was cloned into a pYES2 vector between the *Eco*RI and *Hin*dIII sites. For the confirmation of complementation, the colonies obtained after transformation were dotted with increasing dilutions on –U plates and kept inverted at 30°C (permissive temperature for growth of the yeast *sti* mutant) and 37°C (restrictive temperature for growth of yeast *sti* mutant) for 3 days for the growth of the yeast colonies.

### Subcellular Localization and BiFC Assay

For localization studies, the presence of NLS was predicted using the cNLS mapper online tool (Kosugi et al., 2009). Since it has also been reported that STI localizes to ER, the presence of an even ER signal peptide was therefore also checked in the protein sequences (Chen et al., 2010). Both NLS and ER signal peptides were found in all the STI members (Supplementary Figure S3). To further confirm the localization pattern of these members, the complete ORF sequence along with the signal peptides were cloned for subcellular localization in onion peel. The genes were first cloned in pENTR-topo vector and then mobilized into the destination vector, i.e., pSITE-3CA, pSITE-nEYFP, and pSITE-cEYFP, under a CaMV35S promoter. The ORF of all the *TaSTI* members were fused in frame with the C terminal of YFP. Onion epidermal cells were used for bombardment by using the PDS-1000 bombardment system (Bio-Rad, Canada) at a pressure of 1100 psi with gold particles coated with plasmid constructs (Lee et al., 2008). Transformed onion peels were kept for incubation at 27°C for 16 h in dark condition, and fluorescence was observed in a confocal microscope (Leica, Germany).

### *TaSTI-2A* Cloning and Overexpression in *A. thaliana*

For the generation of *Arabidopsis* overexpression plants, the full CDS of 1.7 kb was amplified with gene specific primers using cDNA as a template isolated from a 10-day-old seedling of *T. aestivum* (PBW343 wheat cultivar). The amplified product was then cloned in an entry vector (pENTR^TM^/D-TOPO) and then in a destination vector pMDC32 under a CaMV35S promoter following the Gateway^TM^ cloning strategy (Directional TOPO cloning kit and LR clonase Enzyme mix II kit, Invitrogen Inc., United States). The GV3101 strain of *Agrobacterium tumefaciens* harboring pMDC32-*TaSTI-2A* was used for transformation in *A. thaliana* through the floral dip method (Clough and Bent, 1998). The T_1_ seeds were selected on MS-agar plates supplemented with 50 μg/μl of hygromycin, and the resistant plants were transferred to pots. Further, these overexpressing transgenics were confirmed by PCR using hygromycin and gene-specific primers. Selected plants were further grown up to the T_3_ homozygous stage. The plants were confirmed by PCR (Supplementary Figure S4).

### *TaSTI-2A* Overexpression in *O. sativa*

Rice (*O. sativa* indica) seeds (variety PB1) were obtained from IARI. Seeds were surface-sterilized by using 0.1% HgCl_2_ (v/v) for 15 min and washed repeatedly with autoclaved sterile water, and then imbibed in water at 28°C for 16 h. Rice transgenics were generated using the protocol described by Toki et al. (2006). Seeds of the Indica rice variety PB1 were grown under light on an NB medium (Himedia labs, cat no. PT107) at 32°C. Co-cultivation was performed with 7-day-old calli with the EHA105 strain of *A. tumefaciens*. These calli were washed after 3 days of co-cultivation and kept on a selection medium containing hygromycin. The positive calli were then transferred to a regeneration medium until plantlets were formed. These plantlets were transferred to rooting medium for 10 days and then on the rice growth medium.

### Histochemical ROS Detection

In order to check the amount of reactive oxygen species (ROS) produced in response to HS in transgenic *Arabidopsis* and WT plants, staining with nitro blue tetrazolium (NBT) was done (Agarwal and Khurana, 2018). For this, 2-week-old seedlings of *Arabidopsis* WT and overexpression transgenics were subjected to HS (42°C for 2 h) after which overnight staining of the plants was done by incubating them in NBT (2 mM NBT powder, 20 mM phosphate buffer). The seedlings were washed with water on the next day and subjected to removal of chlorophyll by dipping them in bleaching solution (ethanol, acetic acid, and glycerol in a ratio of 3:1:1). The plants were then visualized under a bright field light microscope (Leica), and pictures were taken for the comparison of ROS in transgenics and the WT *Arabidopsis* plants after heat stress treatment.

For rice, 1-month-old plants were taken, and a similar protocol was followed for the comparison of ROS in rice WT and overexpression transgenic lines. NBT staining was done after giving them heat stress.

### Physiological Analysis of Heat Stress in *A. thaliana* and *O. sativa*

#### Photosynthetic Efficiency (Fv/Fm)

Measurements of modulated chlorophyll fluorescence emission from the upper surface of the leaf were made using a pulse amplitude modulation fluorometer (Junior-PAM chlorophyll fluorometer, H. Walz, Germany). Leaves of plants were dark-adapted for 20 min before measuring the induction of fluorescence. Measurements of the PSII function of maximum photosynthetic efficiency (Fv/Fm) was recorded in rosette leaves after stress treatments in at least 10 plants per line viz. WT and transgenics. The same protocol was followed with the rice transgenics.

#### Estimation of Chlorophyll Content

Two-week-old wild-type and transgenic plants were subjected to HS. For chlorophyll estimation, 100 mg of leaf tissue was taken in a tube containing 2.5 ml of DMSO. Tubes were incubated overnight for chlorophyll bleaching. Absorbance was taken at 645 nm and 663 nm in a UV–Vis spectrophotometer (Hitachi U-2810, Tokyo, Japan), and chlorophyll content was estimated accordingly (Arnon, 1949).

#### Membrane Stability Index

For Membrane Stability Index (MSI) analysis, 2-week-old stressed and non-stressed seedlings were used. MSI was determined by measuring electrical conductivity with an EC-meter (Eutech, Singapore); 100 mg of leaf tissue was dipped in 10 ml of double distilled water. The tubes were kept at 30°C for 30 min, and conductivity was measured (C1). The seedlings were then autoclaved for 15 min, and electrical conductivity was measured again (C2) in the supernatant. Cellular injury was determined accordingly (Agarwal and Khurana, 2018).

### Yield Analysis

*Arabidopsis* plants were grown on half-strength MS medium in petri plates at 20°C with 16-h photoperiod in daily cycle. Two-week-old plants were subjected to HS at 42°C for 4 h and kept back to recovery. To check for acquired thermotolerance, 2-week-old plants were subjected to continuous heat stress at 30°C for its growth and development. Different yield parameters were checked by observing the seed weight, silique number, and silique length of three lines, respectively.

## Results

### Identification of *TaSTI* Gene Members From *T. aestivum*

**FIGURE 1:**
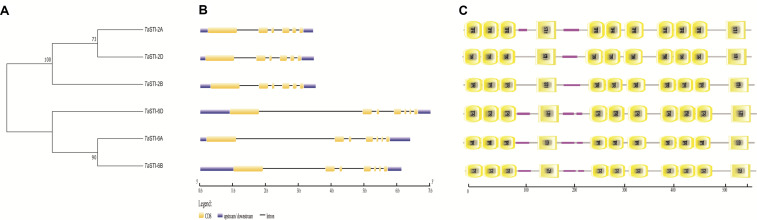
Structural and domain analysis of TaSTI members. **(A)** Phylogenetic relationship between the *TaSTI* gene family members. **(B)** Gene structure analysis of *TaSTI* family members. Exons are depicted using solid boxes and introns are shown using lines. **(C)** TPR and STI domains of all the TaSTI members. Low complexity regions are depicted in solid pink line.

To identify the putative STI members in wheat genome, we searched the database with the known STI proteins as query. In total, we obtained six STI genes in wheat, which were mapped to group 2 and group 6 chromosomes. Therefore, the genes were named according to their chromosomal locations (Table 1). These members were searched for the presence of conserved domains i.e., STI domain and the TPR domain using the CDD search and SMART tools. Domain analysis revealed that all the members had the same number of STI and TPR domains (Figure 1C). The gene structure analysis showed that group 2 *TaSTI* members had a similar organization of introns and exons. On the other hand, all the group 6 *TaSTI* members were found to have longer introns as compared to *TaSTI-2* family members (Figure 1B). *TaSTI-6D* was found to be the longest coding member (2498 bp), whereas *TaSTI-2D* was found to have the shortest coding sequence (2270 bp). These six *STI* members with complete coding sequences were used for further analysis.

**TABLE 1:** *STI* members identified in wheat and their basic information.

Gene name	Gene ID	Nucleotide (bp)	Exon	Intron	Amino acids	Mw (KDa)	Theoretical PI	Chromosomal location
*TaSTI-2A*	TraesCS2A02G386800	2275	6	5	577	64.61	6.77	2A
*TaSTI-2B*	TraesCS2B02G404400	2422	6	5	576	64.38	7.10	2B
*TaSTI-2D*	TraesCS2D02G383600	2270	6	5	572	64.14	6.77	2D
*TaSTI-6A*	TraesCS6A02G238600	2412	7	6	581	65.04	5.78	6A
*TaSTI-6B*	TraesCS6B02G285800	2415	7	6	580	65	5.83	6B
*TaSTI-6D*	TraesCS6D02G221000	2498	7	6	577	64.73	5.78	6D

### Phylogenetic Analysis and Multiple Sequence Alignment of TaSTI Members

**FIGURE 2:**
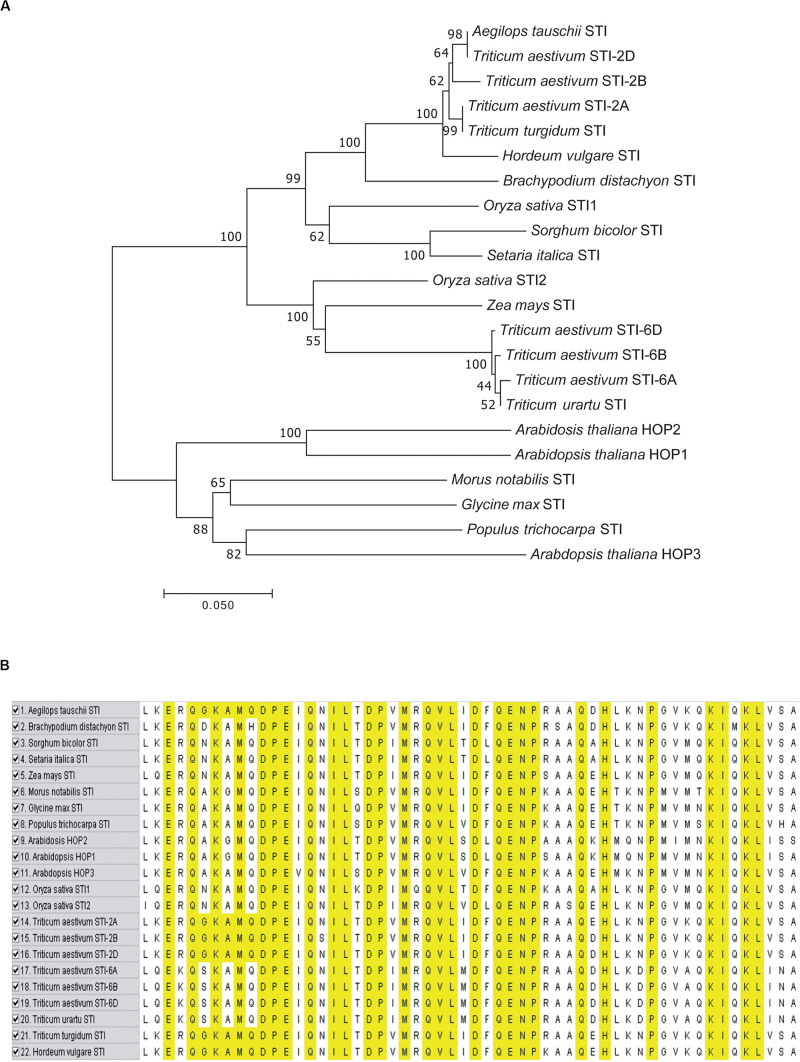
Phylogenetic analysis and sequence alignment of TaSTI members and STI proteins from other plant species. **(A)** Phylogenetic tree based on amino acid sequences depicting the relationship of TaSTIs with other plant STI proteins. **(B)** Amino acid sequence alignment of STI domain of TaSTI members and other STI proteins.

To analyze the evolution of the TaSTIs, we constructed a Neighbor-Joining (NJ) tree based on a total of 22 STI members from different plants (Figure 2A). All the TaSTI members that were present on group 2 chromosomes, were found to belong to a single clade wherein TaSTI-2D was found to be closely related to STI of *Aegilops tauschii*. TaSTI-2A was observed to be close to *Triticum turgidum* STI. However, the TaSTI members that were present on group 6 chromosomes represented a different clade along with *Triticum urartu.* Thus, this suggested the divergent evolution of group 2 and group 6 members in wheat, and probably, these two loci of *STI* genes have existed from the beginning in the *Triticeae* lineage. Further, the multiple sequence alignment of the core STI domain revealed its conserved nature in most of the plants (Figure 2B). Moreover, the three-dimensional structure of TaSTI-2A was predicted and compared with the STI/HOP members of the *Arabidopsis* (Supplementary Figure S2). Interestingly, it exhibited similarity with AtHOP3, which is the one of the HOP members known to function in HS in *Arabidopsis* (Fernandez-Bautista et al., 2018). This in turn suggested that TaSTI-2A might have a role in high temperature stress response and they may even bind to the same client proteins, which are known to bind AtHOP3.

### Promoter Analysis and Expression Profile of *TaSTI* Family Members

The presence of specific *cis*-elements in the promoter region plays an important role in the regulation of gene expression and thus helps in responding to environmental conditions (Walther et al., 2007). Various *cis*-elements were identified in the 2-kb upstream region of all the *TaSTI* gene members by using the PlantCare and PLACE databases. However, in order to assess their role specifically in thermotolerance, the presence of HSE, STRE (stress responsible elements), and CCAATBOX1 elements was analyzed in the promoter regions. HSE forms an essential component of HSR as HSFs bind to these elements to enhance the expression of HSPs and other heat-responsive genes. Similarly, CCAATBOX1 has been known to contribute with HSE elements, and the STRE element has been reported to be involved in various stresses (Khurana et al., 2013). Moreover, it has been documented that the deletion of these motifs in the promoter of *TaHSP26* gene results in the decrease of promoter activity (Khurana et al., 2013). In the case of *TaSTI* gene family members, it was observed that group 2 *TaSTI* members found to possess more HSE elements in their URR as compared to group 6 *TaSTI* members (Supplementary Figure S1). Among the group 2 members, *TaSTI-2B* had two HSEs, whereas *TaSTI-2A* and *TaSTI-2D* were found to have only one HSE in their promoter region. Also, the CCAATBOX1 elements were higher in group 2 members (Supplementary Figure S1). Thus, more heat responsiveness of *TaSTI* group 2 members can be speculated from these data.

**FIGURE 3:**
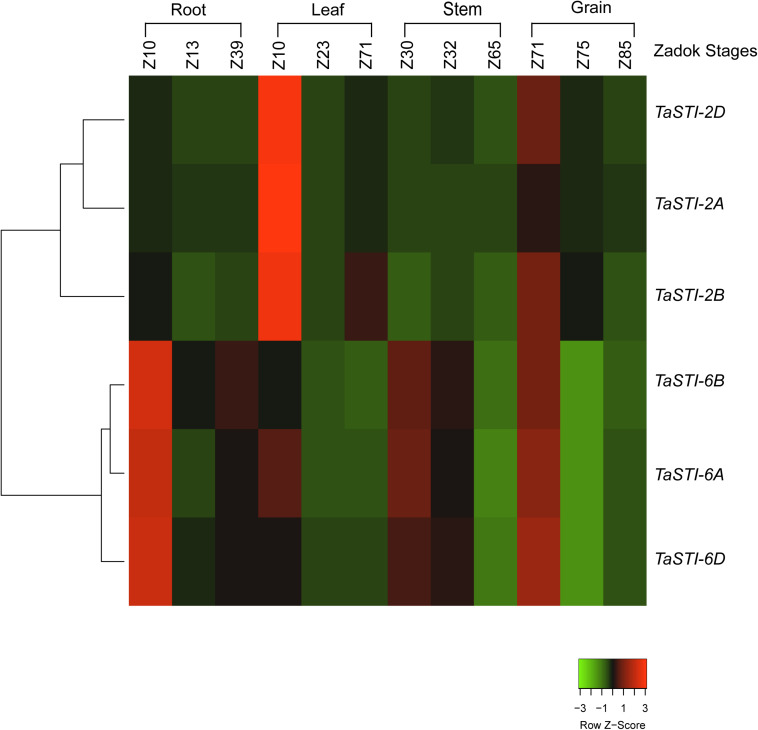
Expression analysis of *TaSTI* family members in different developmental stages of wheat. Heat map showing the relative expression profile of the *TaSTI* family members across the various zadok stages of wheat.

To investigate the functional role of *TaSTI* members, it was important to analyze their expression profile in wheat. For this purpose, the wheat expression database, which comprises RNA-seq data, was used (Borrill et al., 2016). Also, this database offers a method to employ all the published resources in a more meaningful and customizable manner. Thus, we used this database to study the expression pattern and clustering of the identified *STI* members. TPM values of different developmental stages, namely, root, leaf, stem, and grain were analyzed, and it is shown in the form of clustered heat maps (Figure 3). A tissue preferential expression pattern was observed, wherein group 2 *TaSTI* members had higher expression in leaves whereas group 6 *TaSTI* members had higher expression in roots. This indicated that these *STI* members apart from providing protection against HS might have a developmental role in plants.

**FIGURE 4:**
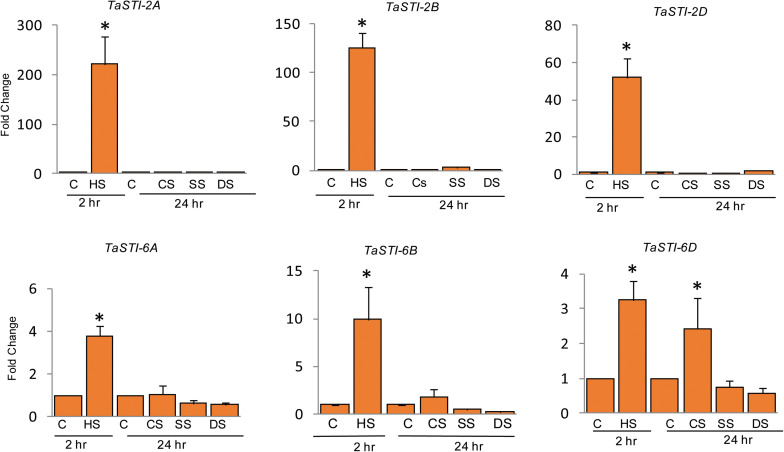
Expression analysis of *TaSTI* family members by real time qPCR. Ten-day-old seedlings of PBW343 were subjected to different abiotic stresses [C-control, HS-Heat stress (42°C for 2 h), CS-cold stress (4°C for 24 h), SS-Salt stress (200 mM NaCl for 24 h), DS-Drought stress (200 mM Mannitol for 24 h)]. Relative fold change was calculated and *TaActin* was used as a housekeeping gene. Graphs were plotted using three biological and three technical replicates. Error bars indicate values ± SD. Asterisks on top of the error bars represent the significance levels (Students *t*-test; *p* ≤ 0.05).

To elucidate the role of these family members during different abiotic stress responses, expression analysis was done by quantitative real-time PCR (qPCR) under four different abiotic stresses such as heat, cold, salt, and drought conditions. Ten-day-old PBW343 seedlings were subjected to these abiotic stresses. As shown in Figure 4, these members were highly upregulated by HS and did not show any significant change in the expression under other stresses. Moreover, *STI* members of group 2 chromosome showed higher expression as compared to the members of the group 6 chromosome, which was in accordance to their promoter analysis. An increase of up to 200 folds was observed in the expression of *TaSTI*-2A under HS conditions.

**FIGURE 5:**
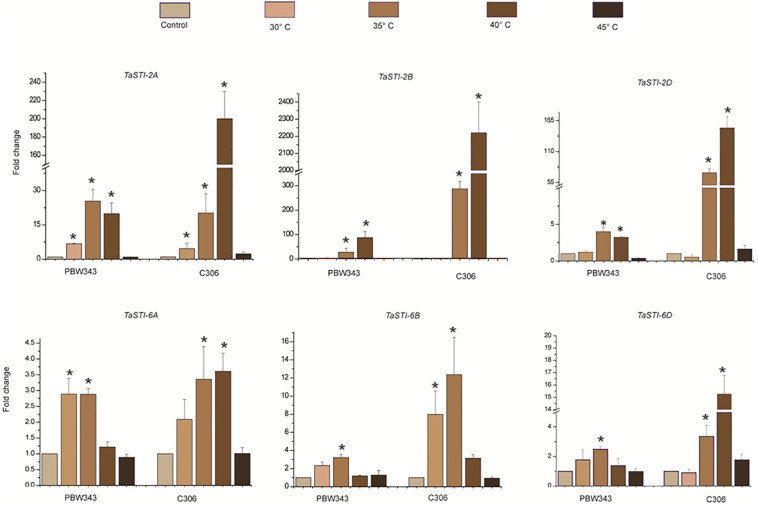
Expression analysis of *TaSTI* family members in PBW343 and C306. Expression was checked in shoot tissue using real-time qPCR. Ten-day-old seedlings were subjected to HS at (30°C, 35°C, 40°C, 45°C) for 2 h. Relative fold change was calculated, and *TaActin* was used as a housekeeping gene. Graphs were plotted using three biological and three technical replicates. Error bars indicate values ± SD. Asterisks on top of the error bars represent the significance levels (Students *t*-test; *p* ≤ 0.05).

A comparative profiling of these members was carried out in thermosensitive and thermotolerant wheat cultivars such as PBW343 and C306, respectively (Hairat and Khurana, 2015), post exposure to increasing temperatures (Figure 5). Higher expression of these members was observed in thermotolerant wheat cultivar C306 as compared to the thermosensitive cultivar PBW343. This indicated a differential regulation of these members in a varietal specific manner and their differential sensitivity to HS. Moreover, in case of group 2 *TaSTI* members, a concomitant increase in expression was observed with increasing temperatures, but their level decreased at 45°C. However, no specific pattern of expression could be observed in the case of group 6 *TaSTI* members. Since *TaSTI*-2A was one of the members, which showed highest induction under HS (Figure 4) and has been earlier identified in our HS library (Chauhan et al., 2011), we selected this gene for further in-depth validation and molecular characterization.

### Subcellular Localization of *TaSTI* Gene Members

**FIGURE 6:**
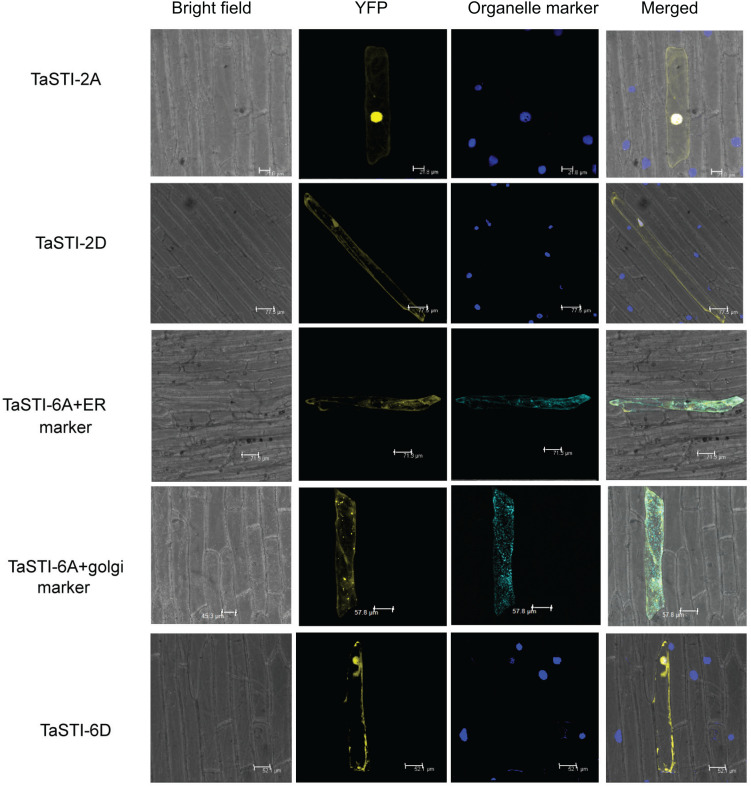
Subcellular localization of selected *TaSTI* genes. CDS of different *TaSTI* genes was cloned in frame with YFP protein and observed under confocal microscope. Different organelle markers were used to locate the gene members in onion epidermis cells. DAPI stain was used to highlight the nucleus along with YFP. ER (Endoplasmic reticulum marker); YFP (yellow fluorescent protein).

Subcellular localization of proteins is an important factor in providing the physiological context of their function. Therefore, in order to determine the subcellular location of *TaSTI* gene members, the pSITE-3CA:*TaSTI* constructs were used to bombard epidermal peels of onion (Figure 6). Two members each from chromosome 2 (TaSTI-2A, TaSTI-2D) and chromosome 6 (TaSTI-6A, TaSTI-6D) were taken for the localization study. YFP signals for TaSTI-2A, TaSTI-2D, and TaSTI-6D were observed in the nucleus and in the cytoplasm. Interestingly, TaSTI-6A was found to accumulate in the cytoplasmic structures resembling ER. To corroborate this result, co-localization was performed with the ER organelle-specific marker. As observed in Figure 6, YFP signals were detected in ER structures that co-localized with CFP-ER. This was in accordance with the result reported by Fernandez-Bautista et al. (2017), wherein AtHOP3 was also found to partially localize to ER.

### Ta*STI-2A* Functionally Complements the Yeast *sti* Mutant

**FIGURE 7:**
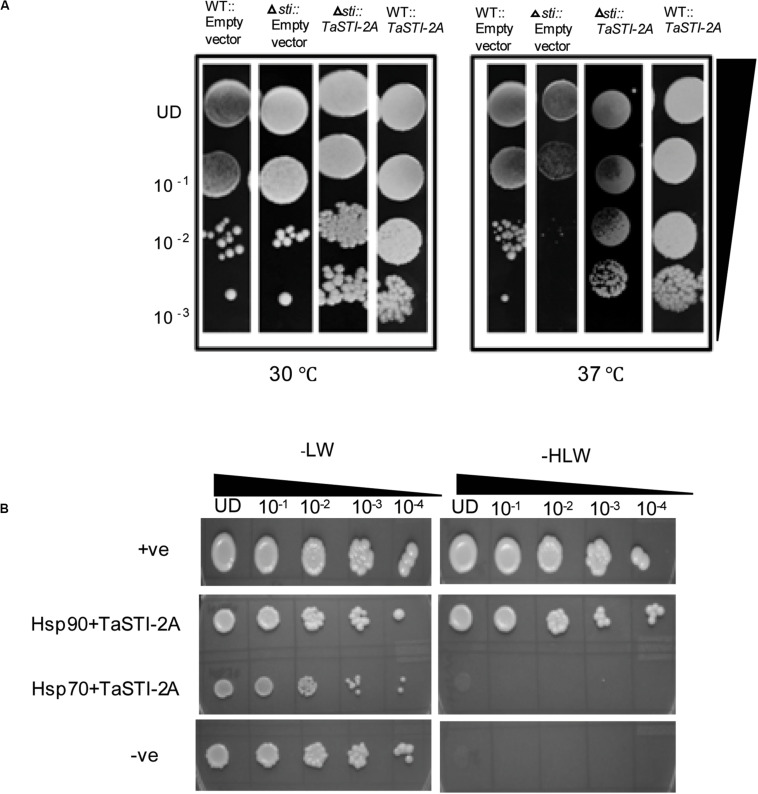
Yeast complementation and yeast two hybrid. **(A)** For the yeast complementation assay, WT and the yeast mutant of the *STI* gene were transformed with empty vector (pYES2) or the fusion construct (pYES2:*TaSTI-2A*). Their growth phenotype was checked at permissive temperature (30°C) or conditional temperature (37°C). **(B)** Yeast-2-hybrid assay was performed to check the interaction of TaSTI-2A with TaHSP90 and TaHSP70 proteins. The growth of yeast cells co-transformed with fusion constructs pDEST-GBKT7:*TaSTI-2A*, pDEST-GADT7:*TaHSP90*, and pDEST-GBKT7:*TaSTI-2A;* pDEST-GADT7:*TaHSP70* was analyzed on SD/-Leu/-Trp (-LW) medium and on SD/-Leu/-Trp/-His (-HLW) medium. The growth of co-transformed cells was monitored by a drop assay on media lacking histidine, leucine, and tryptophan (-HLW) along with 0.5 mM 3-aminotriazole (3AT).

To test the functionality of *TaSTI*-2A, a full-length gene was subcloned into the pYES2 vector, and the construct was transformed into the yeast *sti* mutant (Thermo Scientific). The *STI* mutation in yeast causes mild growth defect at 37°C, which is the restrictive temperature for the growth of yeast (Fernandez-Bautista et al., 2018). The colonies obtained after transformation were thus dotted on SD/-Ura plates and incubated at 30 and 37°C. Vector-transformed WT yeast (AH109, Clontech) was found to grow at both 30 and 37°C, although the colony diameter at 37°C was less than at 30°C (Figure 7A). The vector-transformed mutant, however, showed less growth at 37°C as compared to that at 30°C (Figure 7A). The complementation of the *TaSTI-2A* gene in the mutant improved its growth at 37°C (Figure 7A). Moreover, the overexpression of the gene in the WT resulted in more vigorous growth of even the WT yeast in the restrictive growth condition (Figure 7A). The *TaSTI*-2A gene could thus complement the absence of the yeast *STI* gene in the heterologous system and confer HS tolerance to the mutant. It even provided a growth advantage to WT yeast under the HS condition.

### *In vivo* Interaction of *TaSTI* Members With *TaHSP90* and *TaHSP70*

**FIGURE 8:**
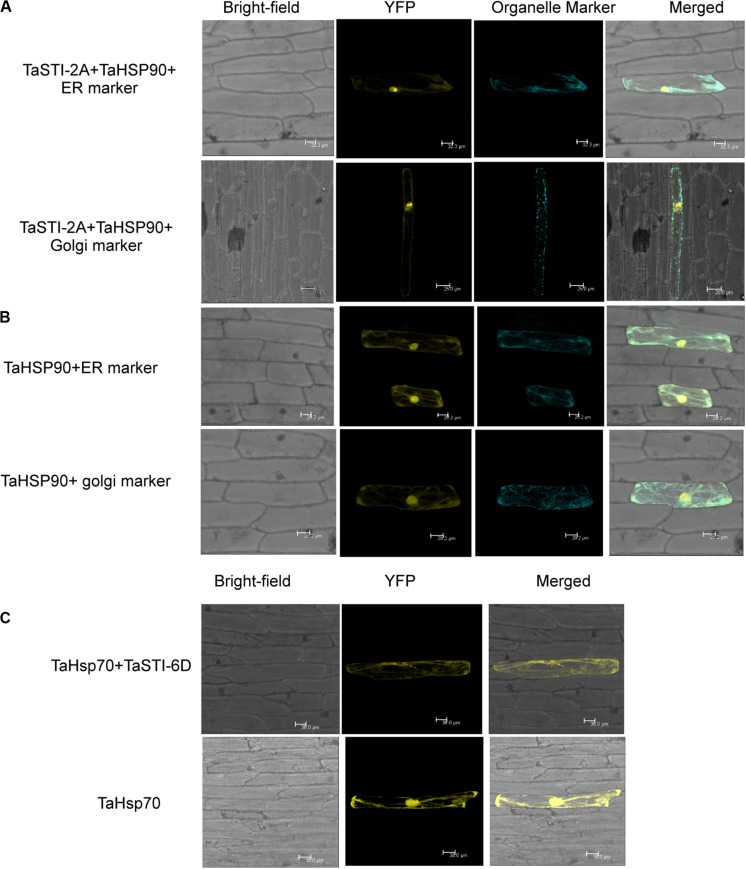
Interaction of TaSTI-2A and TaSTI-6D with TaHSP90 and TaHSP70, respectively. **(A)** BiFC assay was performed to study the interaction between TaSTI-2A and TaHSP90. Transient expression was checked in onion epidermal cells. The localization of the BiFC signal was compared with the ER and Golgi marker localization. Positive interaction could be detected in ER and Golgi bodies along with the nucleus. **(B)** Subcellular localization of the selected TaHSP90 protein. CDS of *TaHSP90* gene was cloned in frame with YFP and observed under confocal microscope. Organelle markers were used to locate the protein in onion epidermis cells. ER (endoplasmic reticulum marker); YFP (yellow fluorescent protein). **(C)** TaSTI-6D and TaHSP70 interaction was observed in cytoplasm using the BiFC assay. For subcellular localization of TaHSP70, CDS of *TaHSP70* gene was cloned in frame with YFP, and transient expression was checked in onion epidermal cells under a confocal microscope.

Earlier studies have reported that STI is a co-chaperone of HSP90 and HSP70; therefore, it forms a protein complex in order to assist these chaperones to carry out the folding of client proteins (Chen and Smith, 1998; Scheufler et al., 2000). Therefore, we examined the interaction of TaSTI-2A with TaHSP90 and TaHSP70 in yeast by using the yeast two-hybrid system. The interaction was found between TaSTI-2A and TaHSP90. All colonies, which had appeared on the −LW media, showed vigorous growth even at 10^–3^ dilution on −HLW media at 0.5 mM 3AT, indicating the interaction to be specific and strong. Interestingly, as shown in Figure 7B, TaSTI-2A did not interact with TaHSP70 in the yeast system. This interaction was further validated by BiFC analysis (Figure 8A). Positive BiFC signals were observed in the nuclei as well as in the cytoplasmic structures of the onion epidermal peels. Further, co-localization of the BiFC signals with the ER and Golgi marker also confirmed that the interaction was occurring in the ER and Golgi complex, apart from the nucleus. This could be further explained by the fact that TaHSP90 localized ubiquitously in the cell (Figure 8B). Since the TaHSP90 protein was found in the ER, Golgi, and nucleus, it is therefore probable to find the interaction between the two in these organelles.

Interaction of TaSTI-2D, TaSTI-6A, and TaSTI-6D was also checked with TaHSP90 and TaHSP70 by using BiFC analysis. Surprisingly, interaction between TaSTI-6D and TaHSP70, occurring specifically in the cytosol, was observed. This unique interaction could be again explained by the ubiquitous distribution of TaHSP70 throughout the cell (Figure 8C).

### Overexpression of *TaSTI*-2A Enhanced Heat Tolerance in *A. thaliana*

**FIGURE 9:**
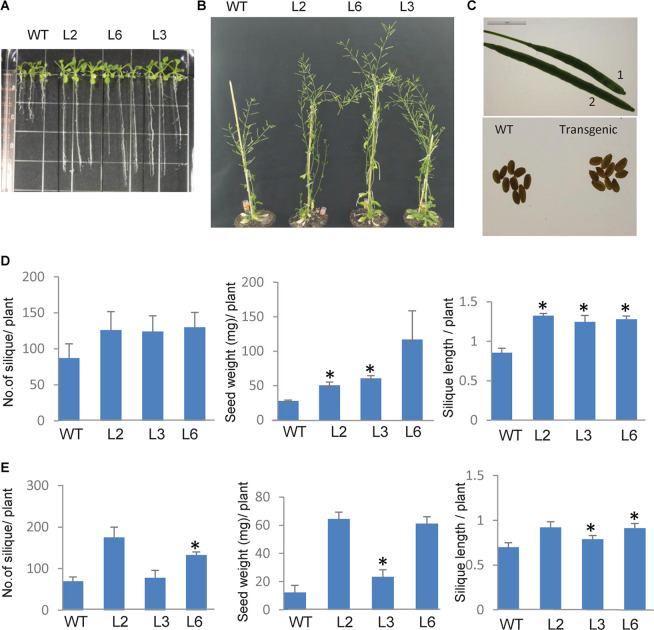
Phenotype of *TaSTI* overexpression transgenic plants of *Arabidopsis thaliana.* Two-week-old plants were analyzed under HS. **(A)** Transgenics showed increased root length in comparison to WT. **(B)** Overexpression line showed better plant growth, **(C)** seed, and silique size (1:WT, 2:OE). **(D)** Graphical representation of silique number, silique length, and seed weight in transgenics and WT at 42°C for 4 h and **(E)** mild heat stress i.e., plants were grown at continuous 30°C. Asterisks on top of the error bars represent the significance levels (Students *t*-test; *p* ≤ 0.05).

To elucidate the functional role of *TaSTI-2A* in plants, overexpression transgenic lines of *Arabidopsis* were generated, and the overexpression in these lines was confirmed using hygromycin-specific PCR and real-time PCR (Supplementary Figure S4). Since this gene was highly upregulated by heat, we therefore observed the growth of the *Arabidopsis* overexpression lines under both basal HS and acquired HS conditions. For basal HS, 2-week-old transgenic plants were subjected to 42°C for 4 h and then returned to their original growth conditions for recovery. After a 15-day recovery period, transgenic plants revived earlier and showed faster and more robust growth as compared to WT (Figure 9B). Also, the transgenic lines showed increased plant height, silique size, and silique number per plant, with respect to WT (Figures 9B,C). Yield parameters like silique number, seed weight, and silique length were found to be better in transgenics as compared to WT (Figure 9D). For acquired thermotolerance, 2-week-old overexpression lines were grown continuously at 30°C. In this case also, transgenic plants performed better than the WT as displayed by their higher number of siliques per plant, silique length, and silique weight (Figure 9E).

As the roots of the plants are equally sensitive to HS as shoots (Giri et al., 2017), root elongation assays were therefore also done in order to check the measure of thermotolerance in overexpression plants. Seven-day-old seedlings were subjected to HS conditions, and it was observed that, in comparison to WT plants, the transgenic plants had longer roots (Figure 9A).

**FIGURE 10:**
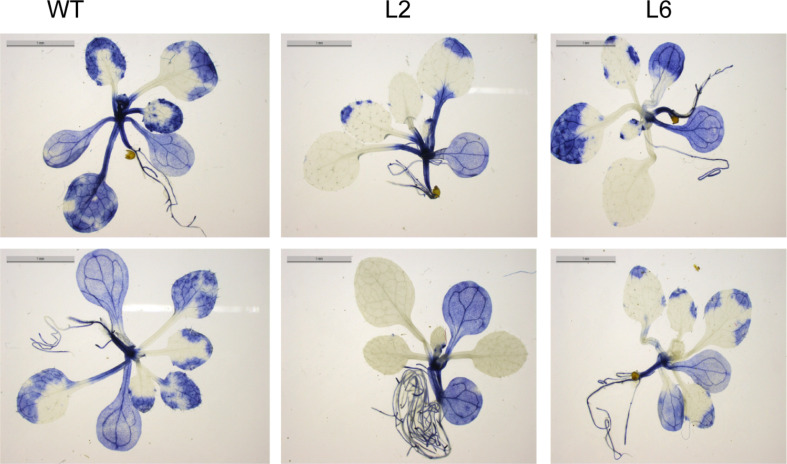
NBT staining. Two-week-old seedling of WT and transgenics were given heat stress at 42°C for 2 h and were then stained with NBT to analyze the superoxide anion accumulation after the stress treatment.

It is well documented that HS accelerates the ROS accumulation in plants and thus leads to oxidative damage (Suzuki and Mittler, 2005). Therefore, the levels of ROS were checked in both WT and the *TaSTI-2A* overexpression lines after HS conditions. The level of super oxide ions as measured by the NBT staining was found to be lower in the transgenic lines in comparison to the WT (Figure 10). Moreover, in all the aspects among the three transgenic lines L2, L6 performed better than L3 which could be justified by the better ectopic expression of *TaSTI-2A* in the L2 and L6 lines (Supplementary Figure S4). Therefore, these two lines were taken for the further physiological assays.

**FIGURE 11:**
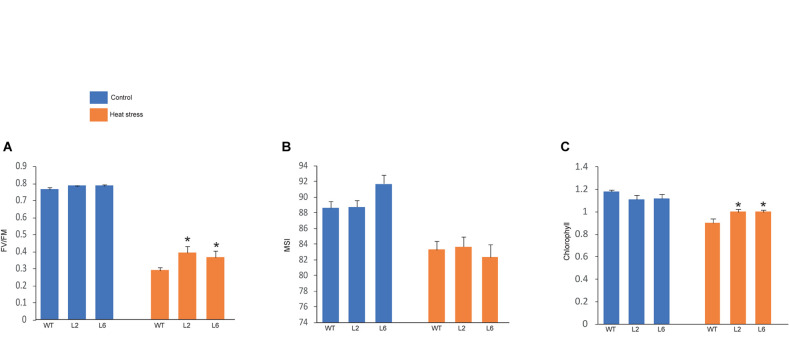
Graphical representation of physiological assays. **(A)** Photosynthetic efficiency, **(B)** cellular membrane stability, **(C)** chlorophyll levels of wild-type (WT) and transgenic lines under control and high temperature stress conditions. Error bars indicate values ± SD. Asterisks on top of the error bars represent the significance levels (Students *t*-test; *p* ≤ 0.05).

The leaf photosynthesis is highly sensitive to high temperature stress (Law and Crafts-Brandner, 1999). Among the whole photosynthetic machinery, photosystem II (PSII) is the most heat susceptible (Havaux, 1992). Therefore, the effect of HS was investigated by measuring the maximum photosynthetic efficiency (Fv/Fm) of PSII. Fv/Fm of transgenics were found to be higher under HS as compared to WT (Figure 11A). The chlorophyll content was also analyzed as chlorophyll synthesis is known to be receptive toward stress and serves as a good indicator (Tewari and Tripathy, 1998). Chlorophyll content (i.e., total chlorophyll content) was found to be higher in transgenics than in WT after stress (Figure 11C). Similarly, membrane stability, which is a measure of ion leakage from the tissue, was used to analyze the damage caused to the members due to HS (Niu and Xiang, 2018). However, no significant difference was found in membrane stability between the WT and transgenic plants (Figure 11B).

### Transcription Profiling of Stress Marker Genes

**FIGURE 12:**
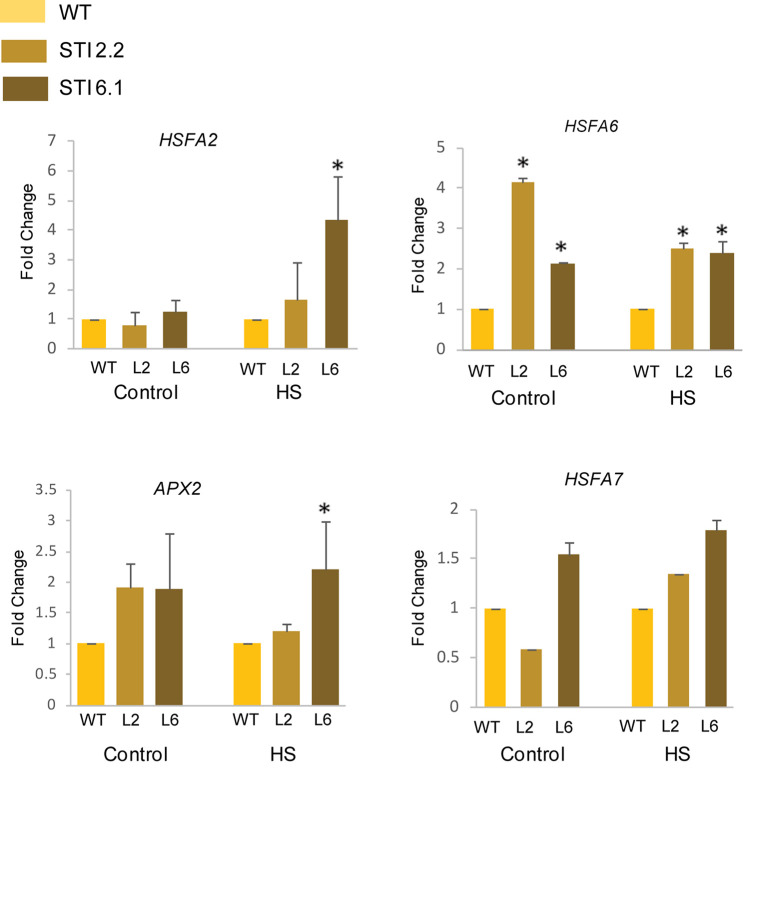
Expression analysis of heat stress marker genes. Transcript analysis of heat stress marker genes in WT and transgenics under control and heat stress. Transcript levels were normalized to WT, and *AtActin* was used as a housekeeping gene. Values represent data from three biological replicates and three technical replicates. Error bars indicate values ± SD. Asterisks on top of the error bars represent the significance levels (Student’s *t*-test; *p*-value ≤ 0.05).

Heat stress transcription factors (HSFs) functions as the major regulators of the HSR as they regulate the expression of various small HSPs and other HSFs (von Koskull-Doring et al., 2007). Interestingly, in case of tomato, it has been shown that HSP70 and HSP90 could regulate the HSR via the interactions with HSFA1, HSFA2, and HSFB1 (Hahn et al., 2011). Thus, in order to assess thermotolerance in *Arabidopsis TaSTI-2A* overexpression lines, we checked the expression of various HSFs in control and HS conditions. It was observed that the relative expression of *AtHSFA2*, *AtHSFA6*, and *AtHSFA7* was found to be higher in overexpression lines than the WT under both control as well as after HS conditions (Figure 12). Apart from these, the expression of antioxidant enzymes were checked as ROS detoxification aids in HS adaptation and in possession of thermotolerance (Yu et al., 2019). The expression of *AtAPX2* was found to be upregulated in overexpression lines under HS conditions in comparison to the WT (Figure 12). Interestingly, even under the control condition, the levels of *AtAPX2* were more in the overexpression lines as compared to WT.

### Phenotypic Analysis of *TaSTI*-2A Transgenic Rice Lines Showed Improved Heat Tolerance

**FIGURE 13:**
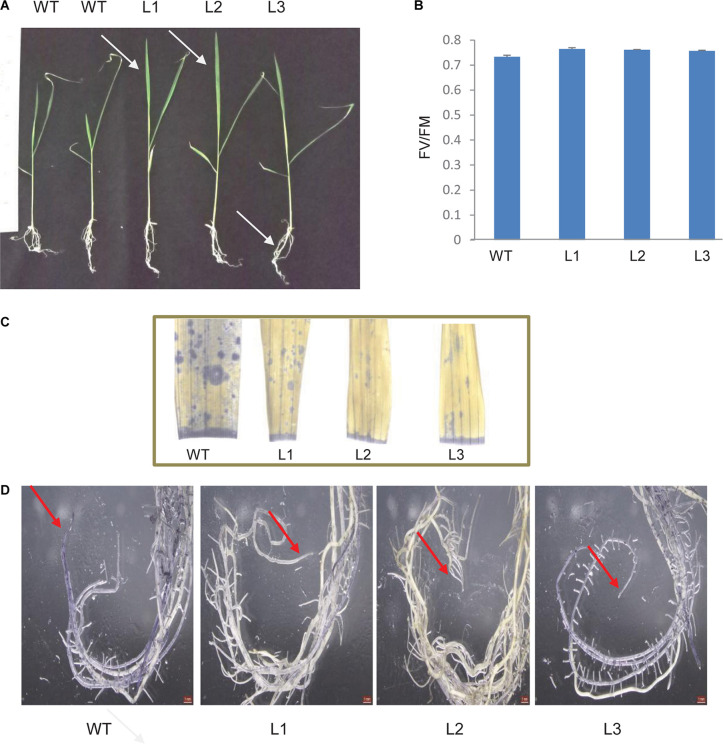
Analysis of rice transgenics under heat stress. **(A)** Five-day-old seedlings were subjected to 42°C for 5 h, and the phenotype was observed after 7 days. **(B)** Photosynthetic efficiency of WT transgenics under HS. **(C)** Localization of superoxide anion in rice leaves and roots by using NBT stain. One-month-old rice plants were subjected to 42°C for 5 h and were then stained with NBT. Blue patches represent the accumulation of ROS in leaves and **(D)** root tissue.

*TaSTI-2A* transgenic rice lines were generated using *Agrobacterium*-mediated transformation, and they were confirmed by PCR (Supplementary Figure S5). Five-day-old transgenic plants that were subjected to 42°C for 5 h followed by 7 days of recovery performed better than the WT. The transgenic plants had better leaf and root growth as compared to the WT (Figure 13A). Also, the photosynthetic efficiency was found to be enhanced in the transgenics (Figure 13B). Furthermore, transgenics in comparison to WT were found to have lesser oxidative load after HS as evident by the NBT staining (Figures 13C,D).

## Discussion

To maintain cellular homeostasis, all eukaryotic cells are equipped with mechanisms to prevent the aggregation of misfolded proteins. HSP90 and HSP70 represent two main chaperone proteins, which play an important role in protein folding, especially under HS conditions (Hahn et al., 2011). However, the binding of different co-chaperone proteins regulates their substrate selection. Although the main chaperones have been deeply characterized in plants (Xu et al., 2012; Usman et al., 2017), our knowledge about the co-chaperone proteins still remains limited. In plants, STI (also known as HOP) serves as one of the co-chaperones for the HSP proteins (Zhang et al., 2003). In the present work, we have identified six members of the *STI* gene family in *T. aestivum*. This number is comparatively higher than the number of *STI* gene family members identified in other plants (Chen et al., 2010; Fernandez-Bautista et al., 2018). The analysis of the STI and TPR domains showed that they share a high similarity with other plant STIs indicating its conserved nature. The members were found to be located to the long arm of group 2 and group 6 chromosomes, and thus, they were named according to their chromosomal locations. Since the redundancy of genes in wheat could be attributed to its hexaploid nature, high similarity was therefore found between these members.

It is noteworthy that the *TaSTI* family members displayed tissue specific transcription pattern, particularly specific to leaves and roots. It is well known that HS not only primarily affects leaf photosynthesis but can also cause damage to root and decrease nutrient uptake (Giri et al., 2017; Wang et al., 2018). Therefore, it is likely that either these members might be involved in protection against HS in these particular tissues or they may be required in developmental pathways in these tissues. In the case of animals, it has already been reported that HOP plays an important role during embryonic development (Baindur-Hudson et al., 2015). Moreover, all the *TaSTI-2* members possessed more heat-responsive elements in their promoter regions and, thus, displayed very high expression under HS. This suggested their indispensable role in QC, which is especially high in HS inside the cells. Also, a differential expression pattern of these *TaSTI-2* members was observed in thermotolerant varieties in comparison to thermosusceptible varieties. These results suggest that the higher expression of these co-chaperone family members might represent one molecular aspect of imparting the tolerant phenotype to the C306 variety.

*STI* members have been previously studied in *Arabidopsis* and in rice, and these members have been found to localize to ER as well as in the nucleus (Chen et al., 2010; Fernandez-Bautista et al., 2018). In accordance to this, our study also displayed that most of the members are localized to the nucleus and the cytoplasm, but only TaSTI-6A was found to reside in the ER and the Golgi complex. Therefore, we postulate that TaSTI-6A might be one of the members, which might help in maintaining QC specifically in ER.

It is well known that STI interacts with HSP70 and HSP90, but interestingly, our work highlights that TaSTI-2A interacts with TaHSP90 not only in the nucleus but also in the ER and Golgi bodies. Thus, we speculate that TaSTI-2A interacts with TaHSP90 in the nucleus and then translocates to the ER. Fernandez-Bautista et al. (2018) also showed similar results in *Arabidopsis* wherein HOP family members localize to the cytoplasm and translocate to the nucleus after the heat treatment (Fernandez-Bautista et al., 2018). Interestingly, TaHSP70 was found to interact with TaSTI-6D (and not with TaSTI-2A) in the cytosol, which in turn highlights the differential role of *TaSTI* family members in HS in wheat. Previous studies in plants have only reported the interaction between HOP3 and BIP proteins (which are ER resident HSP70 proteins) in the ER (Fernandez-Bautista et al., 2017). Taking all these evidences into consideration, we hypothesize that under HS conditions, TaSTI-2A moves form the nucleus to the ER, and TaSTI-6D moves from the nucleus to the cytosol to help in the protein folding response in their respective places. However, further experiments need to be conducted to elucidate the role of these two *TaSTI* family members in HS. Moreover, in this study, only one member of the TaHSP90 and TaHSP70 family was taken for checking the interactions with the TaSTI family members. Therefore, there exists a possibility that the rest of the members of TaSTI may interact with other members of the TaHSP90 and TaHSP70 family, thereby providing each TaSTI protein a specific role inside the cell.

Besides the well-characterized role of *STI* in biotic stresses, their role in response to abiotic stress tolerance remains to be explored. Only a solitary recent report in *Arabidopsis* highlights the role of the HOP family in long-term acquired thermotolerance rather than serving as an adaptor protein (Fernandez-Bautista et al., 2018). Similarly, in the present study overexpression of *TaSTI*-2A in *Arabidopsis* enhances the basal and acquired thermotolerance of the transgenic plants. The overexpression lines showed decreased root growth inhibition, enhanced plant height, high chlorophyll content, and better photosynthetic activity under HS conditions in comparison to WT. The level of oxidative damage to plants after HS was found to be lower in the transgenics. This in turn corroborated with the higher expression of *APX2* in the transgenics, which is a well-known ROS scavenging enzyme and an oxidative stress marker. The higher expression of *HSFA2*, *HSFA6*, and *HSFA7* in the transgenic lines could be one of the reasons for their better performance.

Similarly, in the case of rice, the overexpression of *TaSTI-2A* promoted better growth of the plants after the exposure to high temperatures as seen in their recovery. The transgenics were observed to have lower ROS levels and better photosynthetic efficiency. Interestingly, both in the case of *Arabidopsis* and rice, particularly better root growth of the transgenics was observed after HS. Therefore, it might be speculated that since STI is a co-chaperone, it may bind to different master regulators and thus protect them under HS condition. Overall, it suggests that *TaSTI-2A* helps in imparting thermal stress tolerance and can be considered as a suitable gene to improve crop plants under extreme environmental stress conditions.

In conclusion, among the *TaSTI* gene family members, *TaSTI-2A, TaSTI-2B*, and *TaSTI-2D* were found to be the heat responsive *STI* members under HS conditions in wheat. Overexpression of *TaSTI*-2A in *Arabidopsis* and rice conferred thermal stress tolerance to the transgenic plants. TaSTI-2A showed *in vivo* interaction with TaHSP90 in the nucleus as well as in the ER and the Golgi complex. In the future, it will be of interest to explore how these two proteins interact in the ER-Golgi complex and the functional implications of this interaction. Also, the role of *TaSTI*-6D in HS and the molecular mechanism behind its interaction with TaHSP70 represents an area of future research.

## Data Availability Statement

STI protein sequences of *Arabidopsis thaliana* were taken from Tair database (AT1G12270, AT1G62740, and AT4G12400). One of the TaSTI members was previously identified in the lab from the HS cDNA library (GD189073) which was submitted to the NCBI database (Chauhan et al., 2011).

## Author Contributions

SM, SD, and HS contributed to experimental validation and PK gave the idea, concept, and facilities for the same. All authors contributed to the article and approved the submitted version.

## Conflict of Interest

The authors declare that the research was conducted in the absence of any commercial or financial relationships that could be construed as a potential conflict of interest.

## Supplementary Material

FIGURE S1 Distribution of different heat stress responsive *cis*-acting elements in the 2-kb URR of *TaSTI* gene family members. *Cis-*acting elements were identified using PLACE and PlantCare databases. Different elements are depicted in different colors.

FIGURE S2 Prediction of a three-dimensional structure of STI protein from *T. aestivum* and *Arabidopsis thaliana*. The structures were predicted using the Pyre2 web portal. TaSTI-2A protein was found to be similar to AtHOP3 protein.

FIGURE S3 Depiction of NLS and ER signal motifs. The presence of ER retention signals, dilysine motifs (KKXX or KXKXX), and RXR motif in the TaSTI protein sequences are depicted by the highlighted residues in red color. The presence of bipartite NLS sequences are depicted by the underlined residues.

FIGURE S4 Confirmation of *Arabidopsis* overexpression lines. *Arabidopsis* transgenics for the *TaSTI-2A* gene were confirmed by using **(A)** gene-specific and **(B)** hygromycin-specific PCR. **(C)** Expression profile of *TaSTI-2A* in WT and overexpression transgenic lines of *Arabidopsis*. The transcription level in WT was normalized as 1.0 and the results shown are the means ± SD of at least three independent experiments.

FIGURE S5 Confirmation of *rice* overexpression lines. Rice transgenics for the *TaSTI-2A* gene were confirmed by using a gene-specific forward primer and vector-specific reverse primer.
