# Supplementary figures and images for: Dissecting the Molecular Function of Triticum aestivum STI Family Members Under Heat Stress
Source: Front Genet. 2020 Aug 19;11:873. doi: 10.3389/fgene.2020.00873 (PMC7466592; doi:10.3389/fgene.2020.00873)

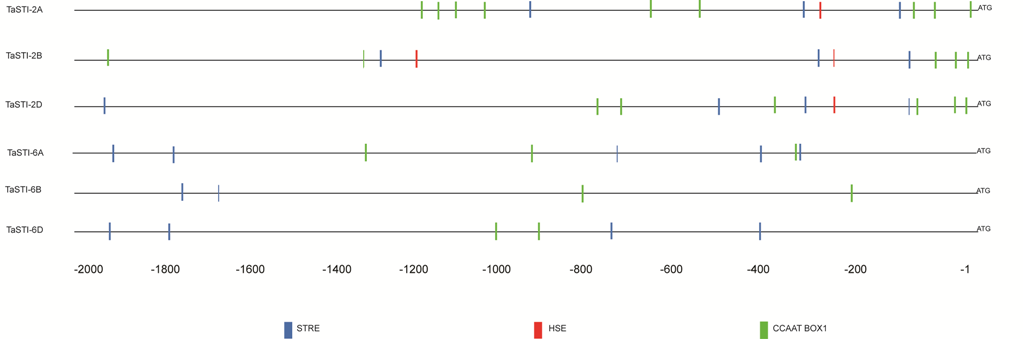

Supplement: FIGURE S1 — Distribution of different heat stress responsive cis-acting elements in the 2-kb URR of TaSTI gene family members. Cis-acting elements were identified using PLACE and PlantCare databases. Different elements are depicted in different colors. [file Image_1.TIF]

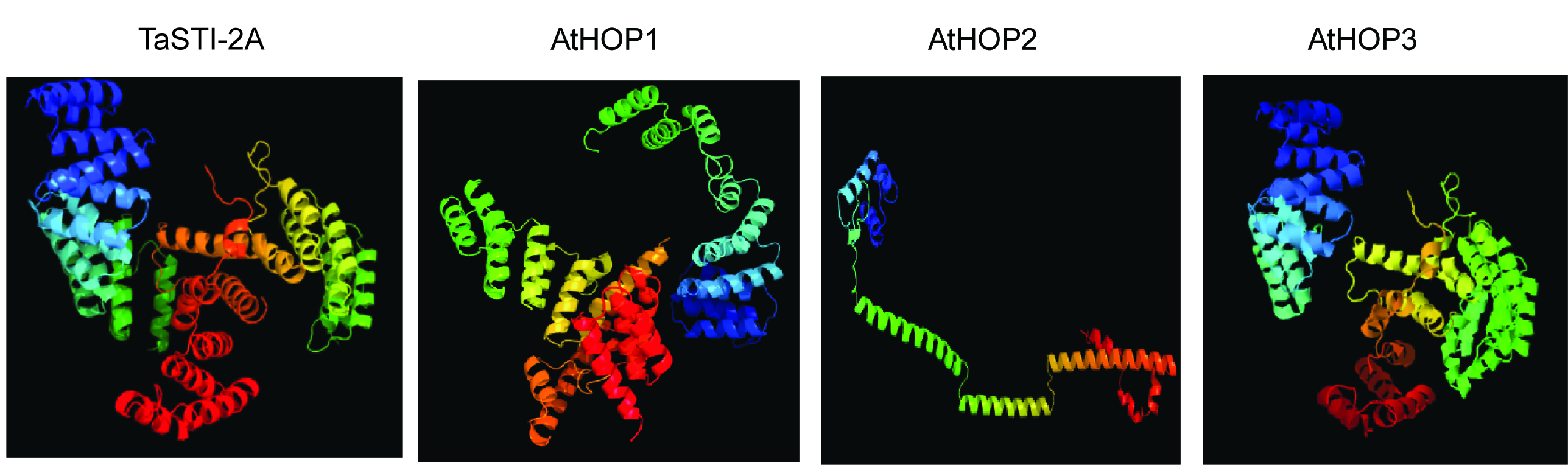

Supplement: FIGURE S2 — Prediction of a three-dimensional structure of STI protein from T. aestivum and Arabidopsis thaliana. The structures were predicted using the Pyre2 web portal. TaSTI-2A protein was found to be similar to AtHOP3 protein. [file Image_2.TIF]

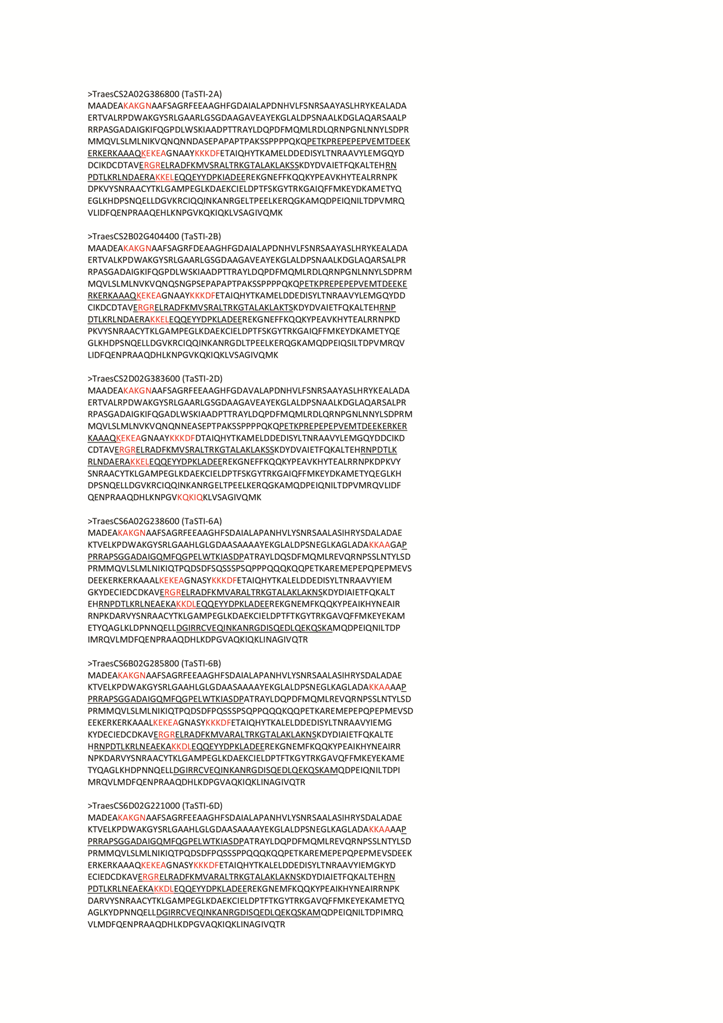

Supplement: FIGURE S3 — Depiction of NLS and ER signal motifs. The presence of ER retention signals, dilysine motifs (KKXX or KXKXX), and RXR motif in the TaSTI protein sequences are depicted by the highlighted residues in red color. The presence of bipartite NLS sequences are depicted by the underlined residues. [file Image_3.TIF]

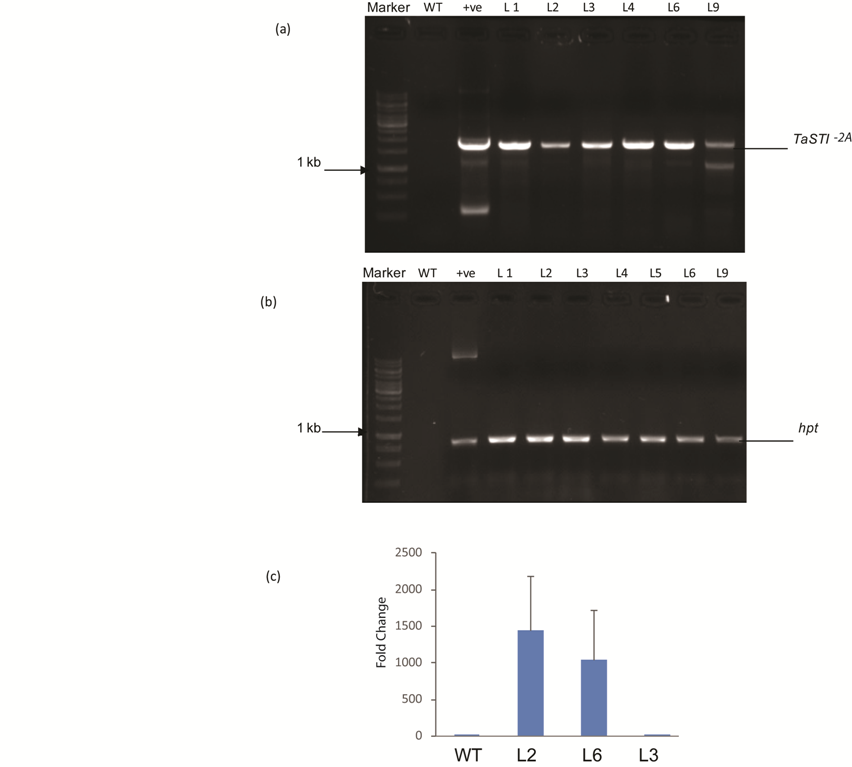

Supplement: FIGURE S4 — Confirmation of Arabidopsis overexpression lines. Arabidopsis transgenics for the TaSTI-2A gene were confirmed by using (A) gene-specific and (B) hygromycin-specific PCR. (C) Expression profile of TaSTI-2A in WT and overexpression transgenic lines of Arabidopsis. The transcription level in WT was normalized as 1.0 and the results shown are the means ± SD of at least three independent experiments. [file Image_4.TIF]

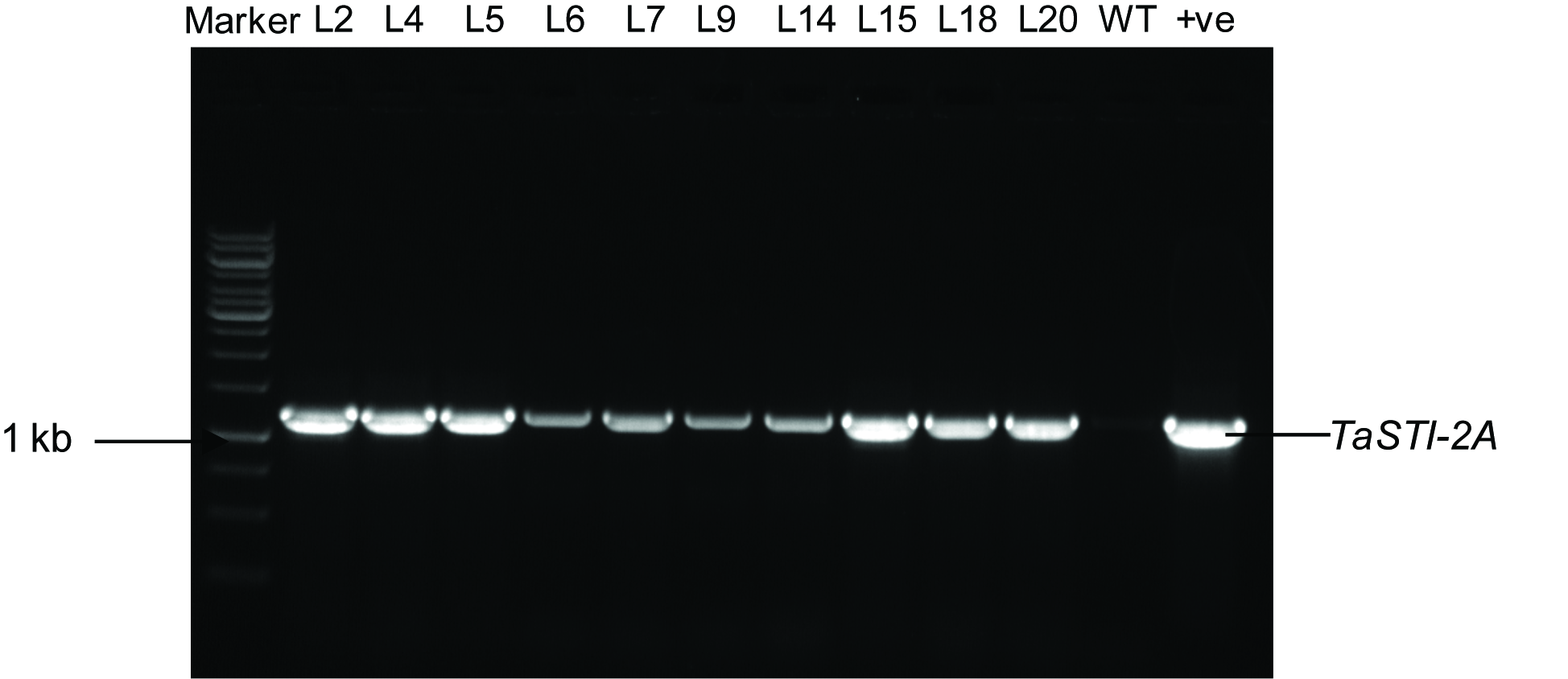

Supplement: FIGURE S5 — Confirmation of rice overexpression lines. Rice transgenics for the TaSTI-2A gene were confirmed by using a gene-specific forward primer and vector-specific reverse primer. [file Image_5.TIF]
